# Injection of Adipose-Derived Mesenchymal Stem Cell-Enriched Adipose Extract for Temporomandibular Joint Osteoarthritis: A Randomised Controlled Trial

**DOI:** 10.1016/j.identj.2026.109604

**Published:** 2026-05-14

**Authors:** Bingshuai Jing, Zerou Zhang, Yaoguang Lv, Shanluo Zhou, Fuwei Liu, Minjie Chen, Zhongcheng Gong, Yunpeng Li

**Affiliations:** a National Clinical Research Center for Oral Diseases, State Key Laboratory of Oral & Maxillofacial Reconstruction and Regeneration, Shaanxi Clinical Research Center for Oral Diseases, Department of Oral and Maxillofacial Surgery, School of Stomatology, The Fourth Military Medical University, Xi'an, Shaanxi 710032, China; b Department of Oral Surgery, Ninth People’s Hospital, College of Stomatology, Shanghai Jiao Tong University School of Medicine, Shanghai, China; c National Center for Stomatology, and National Clinical Research Centre for Oral Diseases, Shanghai Key Laboratory of Stomatology, Shanghai, China; d Department of Oral and Maxillofacial Oncology and Surgery, School/Hospital of Stomatology, The First Affiliated Hospital of Xinjiang Medical University, Urumqi, China; e Stomatological Research Institute of Xinjiang Uygur Autonomous Region, Urumqi, China

**Keywords:** Temporomandibular joint osteoarthritis, Adipose-derived mesenchymal stem cells, Hyaluronic acid, Intra-articular Injection, Randomised controlled clinical trial

## Abstract

**Aims:**

This study aims to compare the efficacy of adipose-derived mesenchymal stem cells-enriched adipose extract (ARDE) with hyaluronic acid (HA) intra-articular injection in the treatment of temporomandibular joint osteoarthritis.

**Methods:**

The randomised controlled trial included patients who did not respond to conservative treatment, were diagnosed with anterior disc displacement without reduction according to the diagnostic criteria for Temporomandibular Disorders, and had a Visual Analogue Scale (VAS) score >4. After joint lavage, the control group received HA injections, while the study group received ARDE injections. The primary outcome included VAS scores for pain, while secondary outcome measures included maximum mouth opening (MMO) and magnetic resonance imaging results. Evaluations were performed at baseline, 1 month, and 6 months postoperatively.

**Results:**

A total of 50 patients were enrolled (25 per group), with comparable baseline characteristics between groups (*P* > .05). Analysis of covariance showed that the least-squares (LS) mean change in VAS score from baseline to month 6 in the ARDE vs HA groups was −4.9 vs −4.3 cm, respectively (LS mean difference, −0.7; 95% CI, −1.1 to −0.2; *P* = .009). For the MMO, the LS mean change was 1.7 vs 1.4 cm, respectively (LS mean difference, 0.3; 95% CI, 0 to 0.5; *P* = .022). Linear mixed-effects models verified significant group and time main effects for VAS score (both *P* < .001) and significant group main effect for MMO (*P* = .009), but no significant group-by-time interaction was found for either indicator (both *P* > .05). Magnetic resonance imaging indicated more significant resolution of preoperative joint effusion in ARDE group (18/22 vs 6/20, *P* < .01).

**Conclusion:**

ARDE injections demonstrate promising potential in relieving pain and improving joint function in patients with temporomandibular joint osteoarthritis, as compared with HA injections.

## Introduction

The temporomandibular joint (TMJ) is among the most complex and functionally intricate joints in the human body, playing a critical role in mandibular movements such as mastication, deglutition, and speech.^1^^,^^2^ Temporomandibular disorders (TMD), induced by multifactorial etiologies including trauma, occlusal disturbances, and immune abnormalities, affect approximately 31% of adults.^1^^,^^3^, ^4^, ^5^, ^6^ Epidemiological studies indicate that temporomandibular joint osteoarthritis (TMJOA), a major subtype of TMD accounting for roughly 55.6% of cases, is characterised by progressive articular cartilage destruction, aberrant subchondral bone remodelling, and chronic synovitis.^7^^,^^8^ Clinically, it manifests as arthralgia, joint clicking, and restricted mobility.^8^, ^9^, ^10^, ^11^ The pathological progression of TMJOA is closely linked to inflammatory responses; pro-inflammatory cytokines such as IL-6, TNF-α, and IL-1β activate signalling pathways that trigger cartilage degeneration and dysregulated bone remodelling.^12^

Therapeutic strategies for TMJOA aim to alleviate pain and restore mandibular function. Conservative and noninvasive modalities are widely accepted as the first-line standard of care, including patient self-management (eg, dietary modification, limiting mouth opening), nonsteroidal anti-inflammatory drugs, occlusal splints, and physical therapy.^1^^,^^6^^,^^11^^,^^13^ When conservative measures fail to control symptoms and imaging indicates progressive degeneration, minimally invasive interventions such as arthrocentesis, lavage, and hyaluronic acid (HA) or platelet-rich plasma injection are considered.^8^^,^^11^ While HA injections restore synovial fluid viscoelasticity and enhance lubrication, their efficacy is often transient, as they neither effectively regulate inflammation nor reverse cartilage degeneration.^11^ Furthermore, repeated injections may increase patient burden and the risk of joint adhesion.^14^^,^^15^ Consequently, there is an urgent need for novel intra-articular injectables capable of modulating the inflammatory microenvironment and arresting degenerative progression.

With rapid advancements in regenerative medicine, bone marrow-derived mesenchymal stem cells (BMSCs) or adipose-derived mesenchymal stem cells (ADSCs) have demonstrated significant potential in treating osteoarthritis.^16^, ^17^, ^18^ A recent meta-analysis of 8 randomised controlled trials (RCTs) reported that mesenchymal stem cells injections significantly improved knee osteoarthritis symptoms, with ADSCs and high-dose regimens showing superior efficacy.^16^ ADSCs offer distinct advantages over bone marrow sources, including abundant availability, ease of harvest, and minimal donor site morbidity. Preclinical studies in the field of TMJ have shown that stem cell therapy provides anti-inflammatory effects, promotes extracellular matrix synthesis, and recruits endogenous stem cells to degenerated cartilage.^2^

In the field of TMJ, clinical research has reported 2 studies involving BMSCs and 3 involving ADSCs, all demonstrating significant improvement effects.^10^^,^^19^, ^20^, ^21^, ^22^, ^23^ Bone marrow aspiration is relatively invasive and has limited availability, which may restrict its widespread application. Among the adipose-derived studies, 2 case reports utilised nanofat and the MyStem kit, respectively, while one RCT employed Lipogems technology and compared it with HA.^10^^,^^19^^,^^20^ However, these approaches are constrained by insufficient cell concentration and a reliance on specialised consumables or equipment.

Our team previously developed an ARDE using mechanical emulsification combined with low-speed centrifugation.^24^ In a rabbit TMJOA model, ARDE promoted cartilage repair and extracellular matrix deposition by inhibiting fibrosis and senescence-related pathways, while reducing the expression of inflammatory cytokines and fibrotic markers. Preliminary clinical data also indicate that ARDE effectively improves symptoms in TMJOA patients. Unlike existing methods that require specialised equipment and yield low cell concentrations, ARDE requires only routine, simple techniques to yield an extract with relatively high stem cell content.^25^ However, further rigorous clinical controlled validation remains necessary. Therefore, this study aims to evaluate the efficacy and safety of arthrocentesis combined with ARDE injection versus HA treatment for TMJOA through an RCT.

## Materials and methods

This research is a prospective, RCT conducted in accordance with the principles outlined in the Declaration of Helsinki concerning medical research involving human subjects. The study has received approval from the hospital’s ethics committee (approval number IRB-YJ-2022033) and is registered with the China Clinical Trial Registry (registration number ChiCTR2300069677). Data regarding the ARDE group's clinical outcomes have been previously reported to validate biological mechanisms.^24^ The present study focuses on the comparative efficacy against the HA control group. Patient enrollment was conducted from 2023 to 2025 and includes participants with unilateral or bilateral conditions, who will be assigned to either the HA group or the ARDE group in a 1:1 ratio based on a computer-generated random number table. All patients had previously undergone conservative treatments (including occlusal splint therapy and muscle function exercises) with unsatisfactory outcomes prior to enrollment. During the study period, all participants continued to receive these conservative treatments to ensure consistency in baseline care across groups. Due to the nature of the interventions (the ARDE group underwent liposuction, while the HA group only received injections), blinding of patients and treating physicians was not feasible in this study, which may introduce potential bias. However, to minimise measurement bias, strict blinding of outcome assessors was implemented. All outcome measurements were recorded by independent researchers who were not involved in the treatment process and were completely unaware of the patients’ group assignments.

### Sample size

The required sample size was determined using PASS software, employing “analysis of covariance (ANCOVA)” (α = 0.05, power = 0.80, R^2^ = 0.3); a minimum of 48 patients was required. To mitigate the impact of potential dropout, the sample size was increased by 10% from the calculated minimum sample size, resulting in a total enrollment of 54 patients (27 per group).

### Inclusion and exclusion criteria

The detailed inclusion and exclusion criteria for patient enrollment are presented in Table 1.

**Table 1:** Inclusion and exclusion criteria.

Inclusion criteria	Exclusion criteria
Patients diagnosed with TMD according to DC/TMD diagnostic criteria	History of infection or trauma in the face
VAS pain score > 4.0	History of neurogenic headache
MMO<3.0 cm	Connective tissue or autoimmune diseases
Patients were diagnosed with ADDwoR based on MRI	Previous history of TMJ injection therapy
Wilkes stages III or IV	History of TMJ surgery
Patients who had already received at least 3 months of conservative treatment (including occlusal splint therapy, pharmacological treatment, and/or physiotherapy) without achieving satisfactory improvement	Contraindications for fat harvesting
Patients in good general health with no systemic diseases such as rheumatism	Unwillingness to participate or incomplete medical history
Ability to cooperate with examinations and complete medical history	

Abbreviations: ADDwoR, anterior disc displacement without reduction; MMO, maximum mouth opening; MRI, magnetic resonance imaging; TMD, temporomandibular disorders; TMJ, temporomandibular joint.

### Surgical steps

#### HA injection

Patients were instructed to repeatedly open and close their mouths to assess the position and movement of the condylar process posterior slope. A puncture point was marked between the joint cavity and the condylar posterior slope (approximately 1 cm in front of the ear). The site was aseptically disinfected with iodine. With the mouth open, the needle was inserted vertically at the marked site, advancing along the posterior slope of the condyle until reaching the joint space, while aspirating to confirm the absence of blood return. Approximately 1.5 mL of 2% lidocaine was then injected, followed by saline lavage, and subsequently, approximately 1 mL of sodium hyaluronate was introduced. Local pressure was applied for 5 to 10 minutes, and patients were advised to avoid hard foods. The HA injections consisted of 2 treatment courses, separated by a 10-day interval, as multiple injections are recommended due to the relatively rapid clearance of HA from the joint space.^26^^,^^27^

#### ARDE injection

**Fig. 1:**
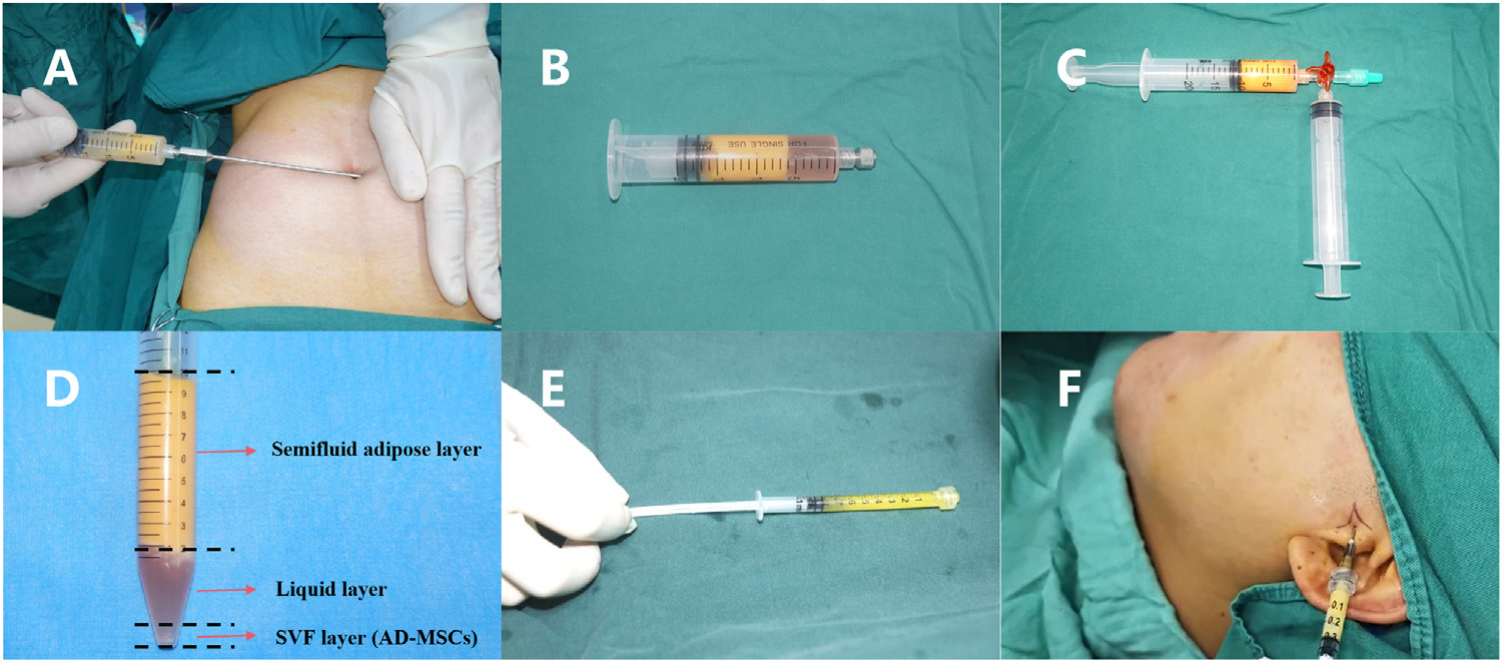
Preparation and joint cavity injection process of ARDE. A, Abdominal adipose tissue was harvested by negative-pressure aspiration. B, The swollen solution was removed by centrifugation. C, The adipose tissue was mechanically emulsified by repeatedly passing it between 2 syringes connected via a three-way valve. D, Subsequent centrifugation separated the emulsion into 3 layers: an upper semi-fluid adipose layer, an intermediate liquid layer, and a lower stromal vascular fraction (SVF) containing ADSCs. E, For clinical injection, the ARDE was prepared by mixing 1 mL of this filtered liquid nanofat with the SVF pellet. F, The prepared ARDE was injected into the upper compartment of the temporomandibular joint.

The area designated for abdominal fat harvesting was marked to assess the volume required. A tumescent solution was created by combining 500 mL of 0.9% saline, 5 mL of 5% sodium bicarbonate, 10 mL of 2% lidocaine, and 0.5 mL of 0.1% epinephrine. A small incision, approximately 3 mm in length, was made in a discreet location near the navel. A 20 mL syringe fitted with a blunt-tipped needle was introduced into the subcutaneous layer, and an appropriate amount of tumescent solution was injected. After a 5-minute pause to enhance vasoconstriction, a lipoaspiration cannula was inserted through the incision into the fat layer, where suction was employed to extract larger fat particles. The harvested fat was centrifuged at 800 rpm for 3 minutes to eliminate residual tumescent solution, resulting in approximately 10 mL of granular fat. Adipose tissue (10 mL) was transferred into two 20 mL syringes connected via a three-way stopcock. Mechanical emulsification was performed by repeatedly pushing and pulling the syringe plungers 60 times. The emulsified tissue was then centrifuged at 1000 rpm for 10 minutes, resulting in the formation of 3 distinct layers: the upper layer consisted of semi-fluid adipose tissue containing lipid fragments, the middle layer contained residual tumescent solution, and the lower layer was composed of stromal vascular fraction (SVF) containing ADSCs. The material from the upper layer was filtered through a sterile 0.5 mm thick nylon mesh to obtain the oily adipose component. This component was then mixed with the SVF from the lower layer to prepare the ARDE formulation. The method for injecting ARDE into the joint cavity is similar to that of HA injection, with an approximately unilateral injection volume of 0.8 ml. For each patient, a 0.5 mL sample of ARDE was collected and sent to the laboratory for cell count analysis (Figure 1 and Supplementary Material 1 for details.) Notably, only one ARDE injection was given, consistent with protocols in previous studies^10^^,^^19^, ^20^, ^21^, ^22^, ^23^ and to avoid the trauma of repeat liposuction within a short timeframe.

### Evaluation methods

All patients underwent preoperative assessment and were followed up at 1 and 6 months postoperatively, with records maintained of any complications encountered. The primary outcome measure was the pain score, evaluated using the Visual Analogue Scale (VAS), where patients indicated their pain sensation on a 10 cm ruler, with 0 representing no pain and 10 representing unbearable pain. Secondary assessments included: (1) Maximum mouth opening (MMO): the vertical distance between the maxillary and mandibular central incisors at the midline when the patient fully opened their mouth; (2) magnetic resonance imaging (MRI) evaluation: assessing the position of the joint disc, presence of joint effusion (high-density images in the joint space on T2-weighted images). Adverse reactions occurring after surgery were documented.

### Statistical methods

Statistical analyses were performed using SPSS version 27.0 (IBM Corp., Armonk, NY). *P*-value < .05 was considered statistically significant. The normality of continuous variables at baseline was assessed using the Shapiro-Wilk test. Normally distributed variables are presented as mean ± SD and were compared between the HA and ARDE groups using independent samples *t*-tests. Non-normally distributed variables are presented as median (interquartile range) and were compared using the Mann–Whitney *U* test. Categorical variables are presented as counts (percentages) and were compared using Fisher’s exact test.

Analysis of covariance (ANCOVA) was conducted to compare the between-group differences in the changes from baseline in the primary and secondary outcomes (VAS score and MMO) at the 6-month follow-up, with baseline values of the corresponding outcomes serving as covariates to adjust for potential confounding and enhance the statistical power of the comparison; the least-squares (LS) means and 95% CI of these outcome changes were calculated for group-to-group comparison. The outcomes, including VAS and MMO, were analysed using Linear Mixed-Effects Models. For each outcome, separate linear mixed-effects models were fitted with the following structure: Fixed Effects: group (HA, ARDE), time (preoperative, 1-month, 6-month as a categorical factor), and their interaction term (group * time). Baseline values of the outcome were included as a covariate to improve model precision.

## Results

**Fig. 2:**
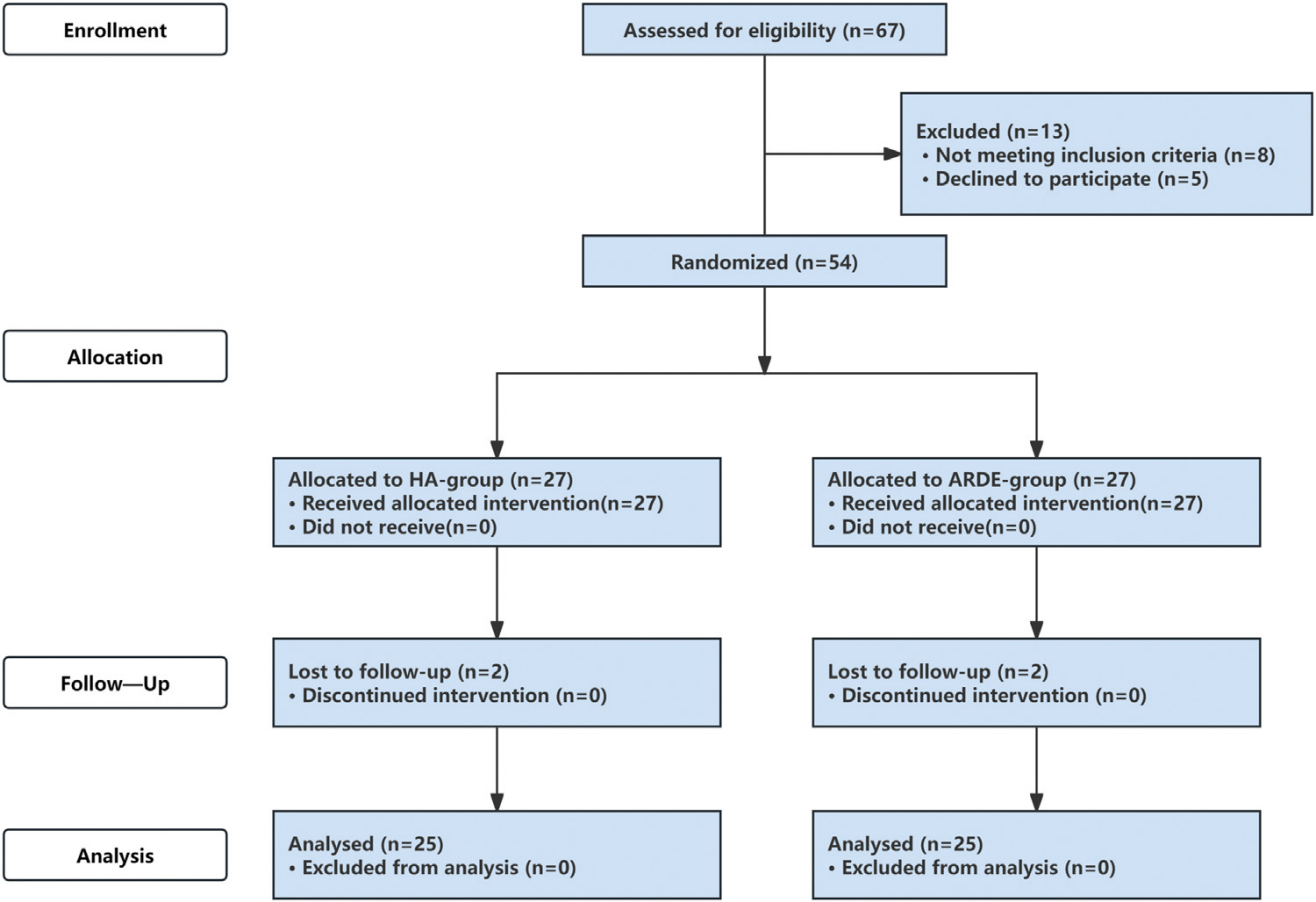
CONSORT flow diagram.

Among the 67 patients screened, 13 were not enrolled, including 8 who did not meet the inclusion criteria and 5 who declined to participate in the study (Figure 2). Two patients in each group were lost to follow-up after intervention. Finally, a total of 50 patients were included in this study, consisting of 25 patients who received ARDE injections and 25 patients who received HA injections. Baseline characteristics of the 2 groups are summarised in Table 2: no statistically significant differences were observed between groups in indicators including age, sex distribution, affected side classification, baseline VAS score, and baseline MMO (all *P* > .05), indicating that the 2 groups had balanced and comparable baseline characteristics after randomisation.

**Table 2:** Patient demographics.

Characteristic		ARDE	HA	*P*-value
Number		25	25	
Age (y), median (IQR)		25 (8)	28 (12)	.16*
Sex, *n* (%)				.75†
	Male	8 (32)	6 (24)	
	Female	17 (68)	19 (76)	
Affected side, *n* (%)				.88†
	Bilateral DDwoR	8 (32)	10 (40)	
	DDWOR + DDwR	9 (36)	8 (32)	
	Unilateral DDwoR	8 (32)	7 (28)	
VAS (cm), Mean ± SD		5.96 ± 0.63	6.06 ± 0.70	.58‡
MMO (cm), Mean ± SD		2.27 ± 0.53	2.36 ± 0.47	.52‡

Abbreviations: ADDwR, anterior disc displacement with reduction; ADDwoR, anterior disc displacement without reduction; ARDE, nanofat enriched with adipose-derived stem cells; HA, hyaluronic acid; IQR, interquartile range; MMO, maximum mouth opening; VAS, Visual Analogue Scale.

⁎ Mann-Whitney *U* test.

† Fisher’s exact test.

‡ Independent samples *t*-tests.

In the ARDE group, nucleated cell counts were determined through cell counting analysis of ARDE samples from all patients. The average total number of cells injected into the joint cavity per patient was 2.22 × 10^6^ cells, with a minimum of 1.73 × 10^6^ cells and a maximum of 2.74 × 10^6^ cells. All patients completed follow-up, and no serious complications, such as infections, fever, or hematoma, were observed during or after the procedures. Although some patients reported increased pain at the injection site 3 days postoperatively, this pain gradually subsided, and by the 1-month follow-up, all participants noted significant pain reduction compared to preoperative levels, with swelling resolving completely within 2 weeks. Additionally, 6 patients in the HA group underwent open surgery for disc relocation due to persistent symptoms following follow-up, while one patient in the ARDE group also required open surgery.

### Primary outcome (VAS score)

**Fig. 3:**
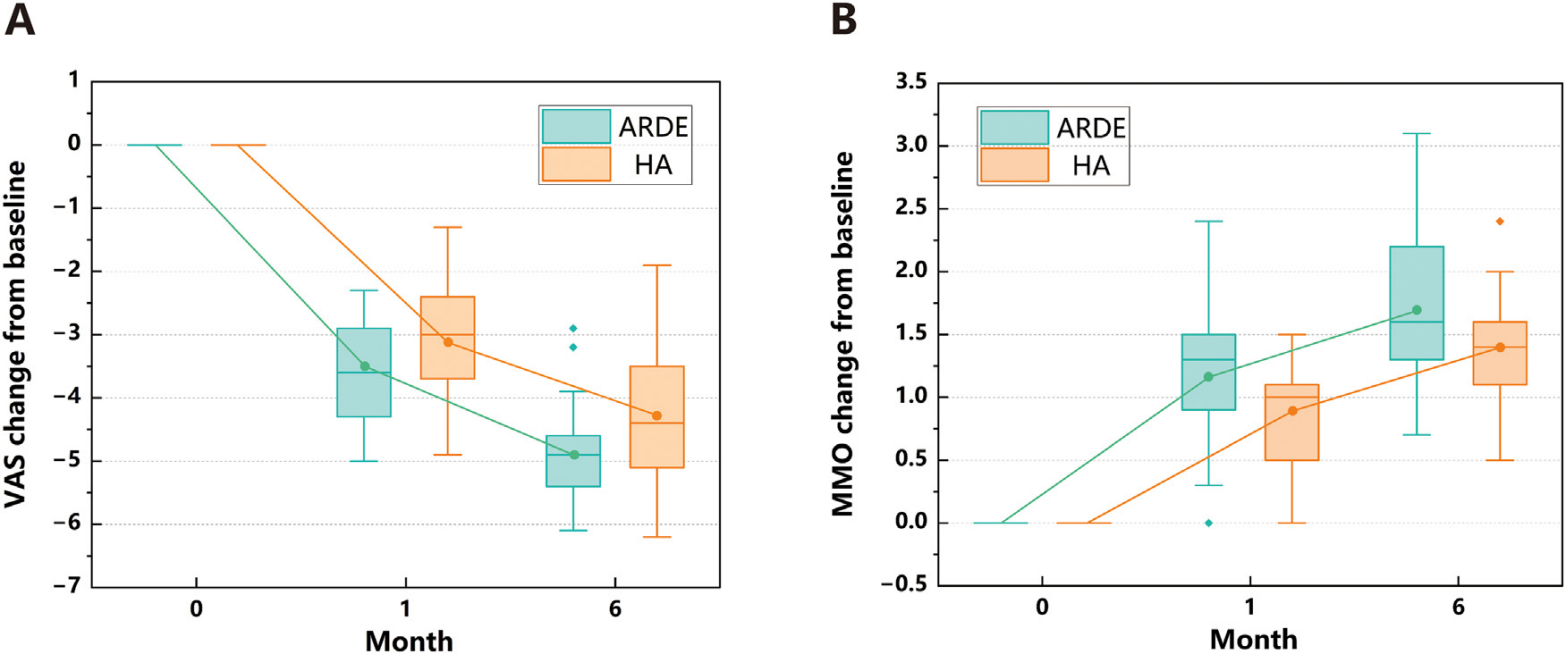
Change from baseline in VAS and MMO scores over time in the HA group and the ARDE group. A, Time-dependent changes from baseline in VAS score; B, Time-dependent changes from baseline in MMO score. This figure depicts the distribution of changes from baseline in VAS and MMO scores across the ARDE and HA groups at different follow-up time points, with 2 panels: (A) VAS scores and (B) MMO scores. The horizontal line inside each box denotes the median of the score changes; the upper and lower boundaries of the box correspond to the interquartile ranges (IQRs); the whiskers extend to the extreme values within 1.5 × IQR; individual data points beyond 1.5 × IQR are presented as outliers; the coloured dots (corresponding to the ARDE and HA groups, respectively) connected by fitted curves represent the least-squares means of the score changes, which were derived from a covariate-adjusted analysis of covariance (ANCOVA) model.

The primary outcome was the change in VAS score, with 25 patients in both the ARDE and HA groups. ANCOVA showed that at 6-month follow-up, the ARDE group had a baseline VAS score of 6.0 ± 0.6 cm (post-follow-up: 1.1 ± 0.7 cm), with an LS mean change from baseline of −4.9 cm (95% CI, −5.3 to −4.6 cm). The HA group had a baseline VAS score of 6.1 ± 0.7 cm (post-follow-up: 1.7 ± 1.0 cm), with an LS mean change of −4.3 cm (95% CI, −4.6 to −4.0 cm). Between-group comparison confirmed a significantly greater improvement in the ARDE group (LS mean difference: −0.7 cm, 95% CI, −1.1 to −0.2 cm, *P* = .009; Figure 3 and Table 3).

**Table 3:** Summary of primary and secondary end points at month 6 in patients treated with ARDE vs HA.

	ARDE			HA			Difference, ARDE vs HA, least-squares mean (95% CI)	*P*-value
	Mean (SD)		Change from baseline, least-squares mean (95% CI)	Mean (SD)		Change from baseline, least-squares mean (95% CI)		
End points	Baseline	Month 6		Baseline	Month 6			
Overall population (*n* = 25 for ARDE; *n* = 25 for HA)								
Primary end points								
VAS (cm)	6.0 ± 0.6	1.1 ± 0.7	−4.9 (−5.3 to −4.6)	6.1 ± 0.7	1.7 ± 1.0	−4.3 (−4.6 to −4.0)	−0.7 (−1.1 to −0.2)	.009
Secondary end points								
MMO (cm)	2.3 ± 0.5	4.0 ± 0.3	1.7 (1.5 to 1.8)	2.4 ± 0.5	3.7 ± 0.5	1.4 (1.3 to 1.6)	0.3 (0 to 0.5)	.022

Abbreviations: ARDE, nanofat enriched with adipose-derived stem cells; HA, hyaluronic acid; MMO, maximum mouth opening; VAS, Visual Analogue Scale.

Data are presented as mean (SD) for baseline and Month 6 values, and least-squares mean (95% CI) for changes from baseline (analysed via analysis of covariance). The “Difference, ARDE vs HA” column reports the least-squares mean difference (95% CI) in end-point changes between groups, with *P*-values indicating statistical significance.

Linear mixed-effects model analysis revealed significant between-group (*F* = 11.366, *P* = .001) and time main effects (*F* = 61.081, *P* < .001) for VAS score, but no significant group-by-time interaction (*F* = 0.277, *P* = .600). This indicated consistent temporal trends in VAS score changes between groups, with superior overall improvement in the ARDE group (Table 4).

**Table 4:** Fixed effects test results from linear mixed-effects model for VAS and MMO.

Outcome measure	Group		Time		Group * Time	
	*F*	*P*	*F*	*P*	*F*	*P*
VAS	11.366	.001	61.081	<.001	0.277	.600
MMO	7.485	.009	0.024	.879	0.009	.923

Abbreviations: MMO, maximum mouth opening; VAS, Visual Analog Scale.

### Secondary outcome (MMO score)

The secondary outcome was the change in MMO score. ANCOVA results showed that the ARDE group had a baseline MMO score of 2.3 ± 0.5 cm (post-follow-up: 4.0 ± 0.3 cm), with an LS mean change of 1.7 cm (95% CI, 1.5-1.8 cm). The HA group had a baseline MMO score of 2.4 ± 0.5 cm (post-follow-up: 3.7 ± 0.5 cm), with an LS mean change of 1.4 cm (95% CI, 1.3 to 1.6 cm). The ARDE group exhibited significantly greater improvement (LS mean difference: 0.3 cm, 95% CI, 0 to 0.5 cm, *P* = .022; Figure 3 and Table 3).

Linear mixed-effects model analysis demonstrated a significant between-group main effect for MMO score (*F* = 7.485, *P* = .009), but no significant time main effect (*F* = 0.024, *P* = .879) or group-by-time interaction (*F* = 0.009, *P* = .923). No notable difference in temporal trends of MMO score changes was observed between groups, with more prominent overall improvement in the ARDE group (Table 4).

### MRI imaging assessment

**Fig. 4:**
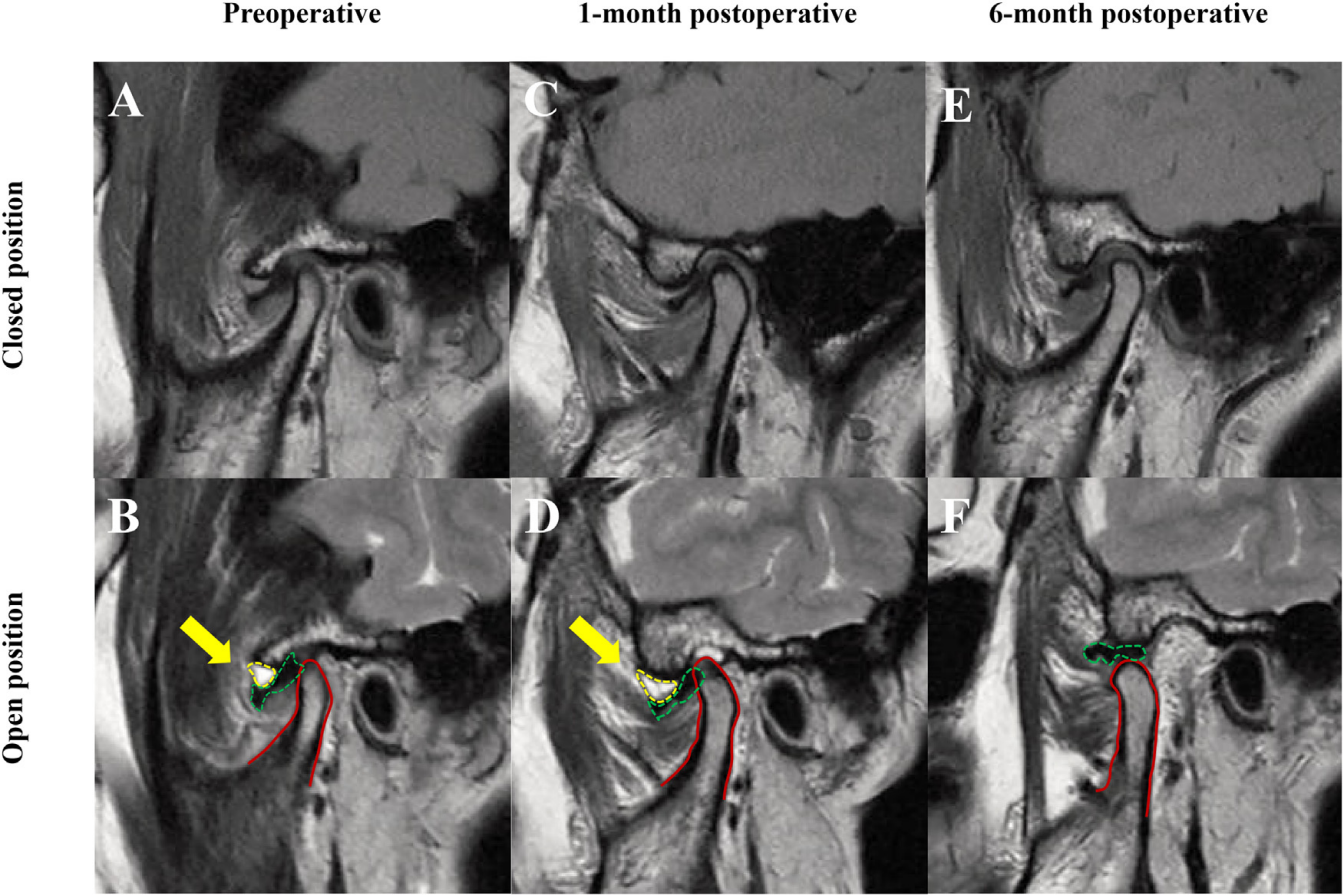
Changes in MRI before and after ARDE treatment. The patient exhibited notable fluid accumulation in the joint cavity prior to surgery, which increased 1 month after the administration of ARDE treatment, potentially due to the injection. However, after 6 months, no significant joint effusion was observed in the joint cavity of the patient (as indicated by the yellow arrow). The patient’s disc position transformed from anterior disc displacement without reduction to anterior disc displacement with reduction (as indicated by the green dashed line).

In the ARDE group, 22 joints demonstrated joint effusion on preoperative MRI, with some patients still exhibiting fat images 1-month post-surgery. By the 6-month follow-up, 18 of these joints had completely resolved effusion. In the HA group, 20 joints had preoperative effusions, with only 6 patients showing resolution. A significant difference in improvement of joint effusion between the 2 groups was noted (*P* < .01). Meanwhile, we also observed that in the ARDE group, 3 joints transformed from anterior disc displacement without reduction to anterior disc displacement with reduction, whereas this phenomenon was not observed in the HA group (Figure 4).

## Discussion

The presence of adipose-derived stem cells in the SVF plays a crucial role in tissue repair due to their pluripotent differentiation potential. ADSCs exert various functions through paracrine mechanisms, including reducing inflammation, promoting cell proliferation, and modulating immune responses.^28^ Autologous fat-derived stem cell-rich injectables are increasingly considered a promising strategy for treating TMD.^10^^,^^29^ In contrast, the ARDE developed in our study addresses this limitation via optimised mechanical emulsification and centrifugation, achieving a higher stem cell yield. Our previous animal experiments and preliminary clinical studies have also confirmed the anti-inflammatory efficacy of ARDE.^24^ To further validate its clinical effectiveness, the present study conducted an RCT comparing ARDE with HA. Consistent with this mechanistic advantage, our results demonstrated favourable safety profiles for ARDE: no severe adverse events were observed postoperatively, with only transient injection-site pain that resolved spontaneously. At the 6-month follow-up, the ARDE group exhibited greater pain relief, improved joint mobility, and more pronounced resolution of joint effusion (evidenced by MRI) compared to the HA group. Although the between-group differences (VAS: 0.7 cm; MMO: 3 mm) were below the commonly cited minimal clinically important difference thresholds (1.0 cm and 5 mm), HA is already an established effective treatment for TMJOA, and ARDE demonstrated slightly greater improvements while offering 2 key advantages: reduced treatment burden and superior joint effusion resolution. Collectively, these findings demonstrate that ARDE, a novel autologous fat-derived injectable, provides enhanced efficacy in the treatment of TMD and highlights its promise as an advanced alternative to conventional HA injections.

Venus et al.^19^ reported in 2019 the use of nanofat for injection therapy in TMJ, but the follow-up time was only 2 weeks, which is relatively short and did not include imaging results. A study using the MyStem system to obtain SVF for treating TMD showed better results than physiological saline joint lavage, though the study group comprised only 4 patients, indicating a limited sample size.^20^ Sembronio et al.^10^ reported in 2021 that the Lipogems technique was employed to acquire micro-fragmented adipose tissue for TMD treatment, using randomised controls against HA treatment. However, none of these studies has clearly reported the total cell or mesenchymal stem cell counts in their adipose extracts. Our study addresses this by demonstrating that the administered ARDE delivered an average of 2.22 × 10⁶ cells per joint injection. Based on recent research findings, we speculate that the number of stem cells injected into the joint cavity is approximately 3.02 × 10⁵ cells.^30^

Lipids play a critical role in joint lubrication, with the hydration lubrication effect of HA and phosphatidylcholine being essential for effective joint function.^31^ Additionally, lipid metabolism abnormalities are characteristic of osteoarthritis and rheumatoid arthritis, as alterations in the lipid composition of synovial fluid and tissue can potentially impact joint lubrication and inflammation.^32^ Previous studies have demonstrated that injecting nanofat coated in polymer microspheres into the joint cavity can effectively enhance the lubrication state of the articular cartilage in animal models.^33^

Injectables containing ADSCs have been reported in multiple studies for treating knee osteoarthritis, significantly alleviating clinical pain symptoms and MRI-confirmed cartilage defects.^18^^,^^34^, ^35^, ^36^ Hudetz et al.^34^ found a significant increase in the proteoglycan content of the extracellular matrix in knee osteoarthritis following the injection of autologous micro-fragmented adipose tissue, suggesting that stem cells may exert effects through paracrine mechanisms. Exosomes secreted by ADSCs can exhibit immunosuppressive and immunomodulatory effects, rendering them promising anti-inflammatory agents for arthritis.^37^ The presence of joint effusion observed in MRI can reflect the inflammatory state of the joint and is associated with the displacement of articular cartilage.^38^^,^^39^ Recent studies have also indicated that joint effusion is an excellent MRI indicator for distinguishing the aetiology of joint pain.^40^ In this study, patients in the ARDE group demonstrated significant improvement in joint effusion compared to the HA group, with a more pronounced reduction in pain. We speculate that stem cells exert anti-inflammatory effects through paracrine action, leading to improvements in joint effusion and consequently accelerating the alleviation of joint pain.

Intra-articular adhesions may significantly restrict joint mobility, which can be identified during arthroscopy and addressed through surgical release.^15^ Liu et al.^14^ found that the incidence of intra-articular adhesions in patients with a history of joint puncture was 69.23%, markedly higher than the 24.36% in those without such a history. These adhesions, which can develop following trauma and bleeding, may result from the formation of fibrous scar tissue.^14^^,^^41^ Patients with prior joint punctures and HA injections may experience increased difficulty in achieving joint gap release during open joint disc anchoring surgery. Notably, injections of adipose-derived products do not appear to induce joint adhesions. In one instance, a patient with pronounced condylar resorption experienced persistent biting pain after receiving adipose-derived injections, accompanied by insomnia and anxiety. MRI revealed anterior disc displacement without reduction on the affected side. This patient ultimately required open surgery, during which no adhesions were observed. This outcome may be partly due to the adipose-derived products maintaining space within the joint cavity, a practice that is not unfamiliar in the treatment of TMD, where adipose flaps serve as barriers to preserve joint gaps, prevent heterotopic ossification, and inhibit re-stiffening of the joint.^42^ Furthermore, bioactive molecules in adipose-derived products may play a role in inhibiting tissue fibrosis.^43^^,^^44^ The improved interincisal opening observed in patients from the ARDE group compared to the control group, along with the transition of patients with anterior disc displacement without reduction to anterior disc displacement with reduction, may also be associated with anti-fibrotic effects.

Several limitations of this study should be acknowledged. First, due to the inherent differences in the interventions (liposuction in the ARDE group versus simple injections in the HA group), blinding of participants and treating physicians was not feasible. This lack of blinding may introduce performance and detection bias, particularly for subjective outcomes such as VAS pain scores. The invasive nature of liposuction in the ARDE group likely heightened patients’ expectations of therapeutic benefit, potentially triggering a powerful “surgical placebo effect” that could have substantially inflated the reported pain relief compared to the HA group, which received only a minimally invasive injection. Although strict blinding of outcome assessors was implemented to minimise measurement bias, the potential placebo effect associated with the surgical procedure cannot be fully excluded. Second, the 6-month follow-up, while adequate for assessing short-term symptomatic improvement, may be insufficient to capture long-term structural changes, particularly in advanced TMJOA (Wilkes stage III/IV). Thus, this study provides preliminary short-term findings; extended follow-up (≥12 months) is warranted to confirm the durability and potential disease-modifying effects of ARDE. Third, the difference in treatment frequency (2 injections in the HA group versus a single injection in the ARDE group) may represent an additional confounding factor. This disparity in procedural burden should be considered when interpreting the comparative efficacy between the 2 groups. Furthermore, while current preliminary studies, including this one, generally indicate that stem cell therapy is effective in TMD, whether it can become the optimal option when conservative treatments fail still requires thorough investigation.^10^^,^^19^, ^20^, ^21^, ^22^, ^23^, ^24^ It is necessary to conduct multicentre, large-sample RCTs in the future to further clarify the efficacy of stem cell therapy. Moreover, similar to ongoing research in knee osteoarthritis, the optimal source and dosage of stem cells for intra-articular injection in TMJOA also warrant further comparative trials.

## Conclusion

The ARDE protocol, derived from autologous fat, demonstrates promising potential in alleviating pain and improving functional impairment in patients with TMJOA, as compared to traditional HA injections. Moreover, it results in substantial improvements in joint effusion. This study underscores the considerable potential of adipose-derived products containing ADSCs for the treatment of TMD.

## Author contributions

Bingshuai Jing and Zerou Zhang contributed equally to this work. Bingshuai Jing and Zerou Zhang: Conceptualisation, Methodology, Validation, Writing – Original draft. Yaoguang Lv, Shanluo Zhou and Fuwei Liu: Data collection, Formal analysis Methodology, Validation, Writing – Review & editing. Minjie Chen and Zhongcheng Gong: Validation, Writing – Review & editing. Yunpeng Li: Conceptualisation, Methodology, Writing – Review & editing, Supervision. All authors gave final approval and agree to be accountable for all aspects of the work.

## Funding

This research was supported by Shaanxi Provincial Health Research and Innovation Platform Construction Plan (2024PT-04), Key Research and Development Program of Shaanxi (2023-GHZD-20).

## Conflict of interest

None disclosed.

## Appendix Supplementary materials

Supplementary material 1: Video of joint cavity irrigation and ARDE injection process. Image, video 1 dummy alt text

## Footnotes

Supplementary material associated with this article can be found in the online version at doi:10.1016/j.identj.2026.109604.
